# Magnetic force microscopy of an operational spin nano-oscillator

**DOI:** 10.1038/s41378-022-00380-4

**Published:** 2022-06-15

**Authors:** Seyed Amir Hossein Banuazizi, Afshin Houshang, Ahmad A. Awad, Javad Mohammadi, Johan Åkerman, Liubov M. Belova

**Affiliations:** 1 Faculty of New Sciences and Technologies, University of Tehran, Tehran, Iran; 2 Materials and Nanophysics, Department of Applied Physics, School of Engineering Sciences, KTH Royal Institute of Technology, 114 19 Stockholm, Sweden; 3 Department of Physics, University of Gothenburg, 412 96 Gothenburg, Sweden; 4 Department of Materials Science and Engineering, KTH Royal Institute of Technology, 100 44 Stockholm, Sweden

**Keywords:** Electronic devices, Electronic properties and materials, Structural properties, Electrical and electronic engineering, Electronic properties and materials

## Abstract

Magnetic force microscopy (MFM) is a powerful technique for studying magnetic microstructures and nanostructures that relies on force detection by a cantilever with a magnetic tip. The detected magnetic tip interactions are used to reconstruct the magnetic structure of the sample surface. Here, we demonstrate a new method using MFM for probing the spatial profile of an operational nanoscale spintronic device, the spin Hall nano-oscillator (SHNO), which generates high-intensity spin wave auto-oscillations enabling novel microwave applications in magnonics and neuromorphic computing. We developed an MFM system by adding a microwave probe station to allow electrical and microwave characterization up to 40 GHz during the MFM process. SHNOs—based on NiFe/Pt bilayers with a specific design compatible with the developed system—were fabricated and scanned using a Co magnetic force microscopy tip with 10 nm spatial MFM resolution, while a DC current sufficient to induce auto-oscillation flowed. Our results show that this developed method provides a promising path for the characterization and nanoscale magnetic field imaging of operational nano-oscillators.

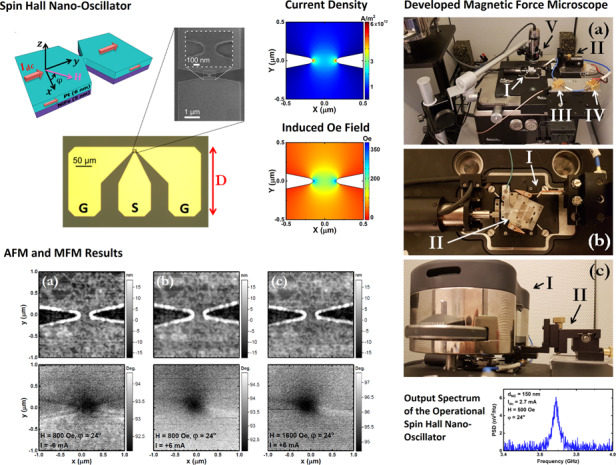

## Introduction

Since the development of magnetic force microscopy (MFM), researchers have explored a variety of techniques for improving its capabilities in terms of studying magnetic microstructures and nanostructures^1–4^. Spin-wave excitations driven by spin-transfer torque (STT)^5–8^ have become an important area of research in the last decade, both from a fundamental point of view and for their potential in applications using spintronic devices, such as spin torque nano-oscillators (STNOs) and spin Hall nano-oscillators (SHNOs)^9^. These nanosized devices have attracted considerable attention for their potential for use in nanoelectronic circuits as high-frequency signal generators^10^, sensors^11,12^, biosensors^13–17^ and, more recently, computing devices^18,19^. Therefore, new methods and techniques are needed to characterize these kinds of devices to gain better insight into their operational specifications.

We fabricated a wide range of STNOs^20–32^ and SHNOs^19,33–40^ with different characteristics. Although our microwave measurements show the expected auto-oscillation outputs from our devices, maps of these devices in the operational state would be useful for real applications. Here, we present a new method for probing the spatial profile of an operational nano-oscillator using magnetic force microscopy (MFM). We employed a very high-resolution MFM system^41^ using specially designed extremely sharp MFM tips^42^ to observe operational nanomagnetic oscillator devices.

## Results and discussion

### Spin Hall nano-oscillator fabrication and characterization

Recent investigations of spintronic devices have shown that the spin Hall effect (SHE) in a nonmagnetic film with strong spin-orbit interaction (such as Pt) can induce pure spin currents that can be used to exert sufficient STT on an adjacent ferromagnetic thin film to drive spin wave auto-oscillations. These kinds of oscillators are called spin Hall nano-oscillators (SHNOs)^33,34,43–45^ and have great potential for application, as they are both easy to fabricate and have reasonably good emission characteristics. The oscillation frequencies and the spin wave modes in SHNOs depend on the applied external magnetic field and the DC current. As a result, the Oersted field (*H*_Oe_) induced by DC current also modifies the effective field landscape in SHNOs and affects some of the main properties of high-frequency emissions. A clear understanding of the magnetization properties of SHNO devices in their operational state is crucial, and the ability to directly measure these properties would be useful; MFM could be used for this.

**Fig. 1:**
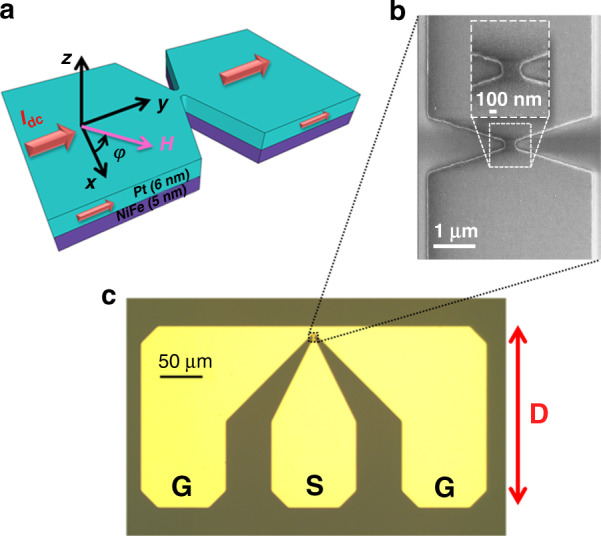
SHNO device. **a** Schematic and **b** SEM image of a nanoconstriction. **c** Optical microscopy image of the final device in a conventional design with top contact coplanar waveguides (with length of D) providing electrical access to the SHNO

We fabricated nanoconstriction-based SHNO NiFe/Pt bilayers with constriction widths ranging from *d*_NC_ = 80 to 300 nm; these are schematically presented in Fig. 1(a), and a scanning electron microscopy image of a nanoconstriction etched out of 5 nm thick permalloy (Ni_80_Fe_20_; Py) and 6 nm thick Pt bilayers magnetron sputtered onto a sapphire substrate is shown in Fig. 1(b).

In our conventionally fabricated devices, shown in Fig. 1(c), ground–signal–ground (GSG) waveguides provide electrical and microwave access to the nanodevice. However, there is limited space under the head of the MFM system employed for this purpose, meaning that short waveguides cannot be used with MFM. We thus fabricated devices with longer waveguides (larger D, as specified in Fig. 1(c)) to ensure that the microwave probe could make stable and safe contact with the waveguides.

**Fig. 2:**
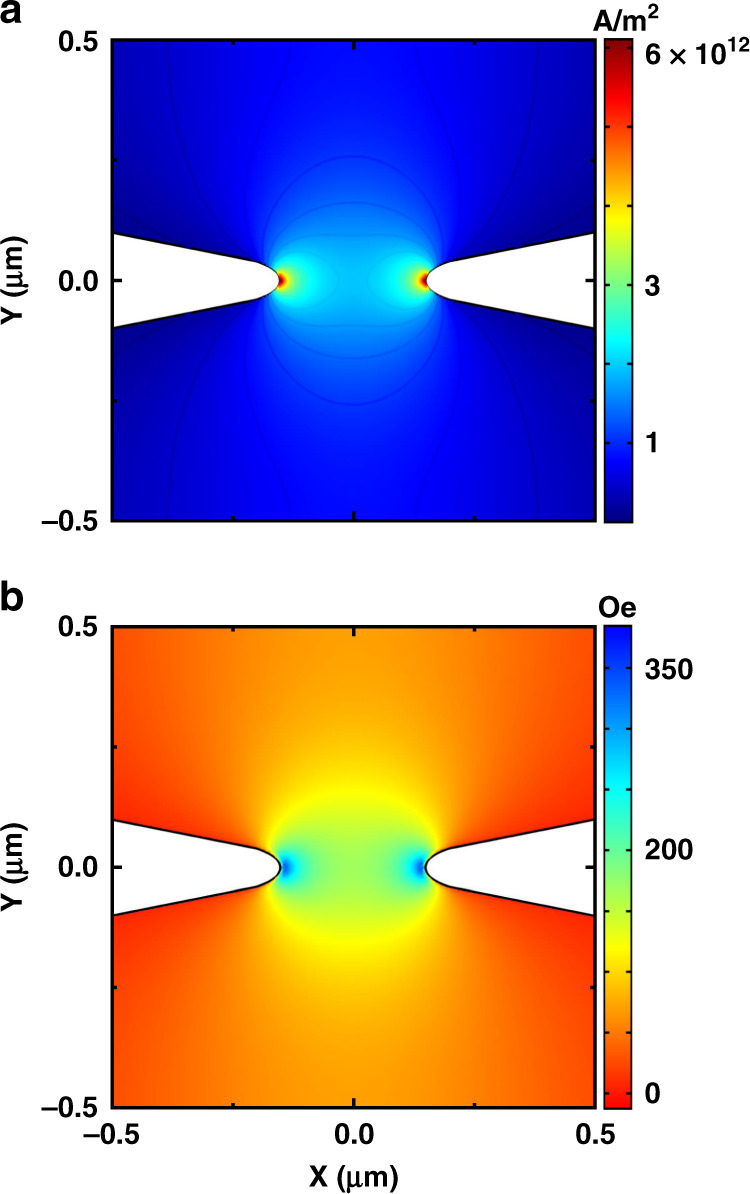
Simulation of current density and induced Oe field in SHNO device. Detailed simulation results of the **a** *y*-component of the current density and **b** *x*-component of *H*_Oe_ at the top of the Pt layer for *I*_dc_ = 6 mA in a nanoconstriction-based SHNO with a constriction width of *d*_NC_ = 300 nm

We investigated how current redistributes and induces *H*_Oe_. We used COMSOL Multiphysics^®^ simulation software with a detailed three-dimensional finite-element model of the nanoconstriction-based SHNO NiFe/Pt bilayers with a constriction width of *d*_NC_ = 300 nm to calculate the current flow and *H*_Oe_ in each layer of the device. We used the nominal dimensions of the real devices and the material properties provided by the program (Fig. 2). In Fig. 2(a), we show the simulation result of the *y*-component of the current density. Figure 2(b) displays the simulated *x*-component of *H*_Oe_ at the top of the Pt layer for *I*_dc_ = 6 mA, where there is a circular area in the middle with a higher *H*_Oe_.

### Electrical microwave spectroscopy using a customized probe station

**Fig. 3:**
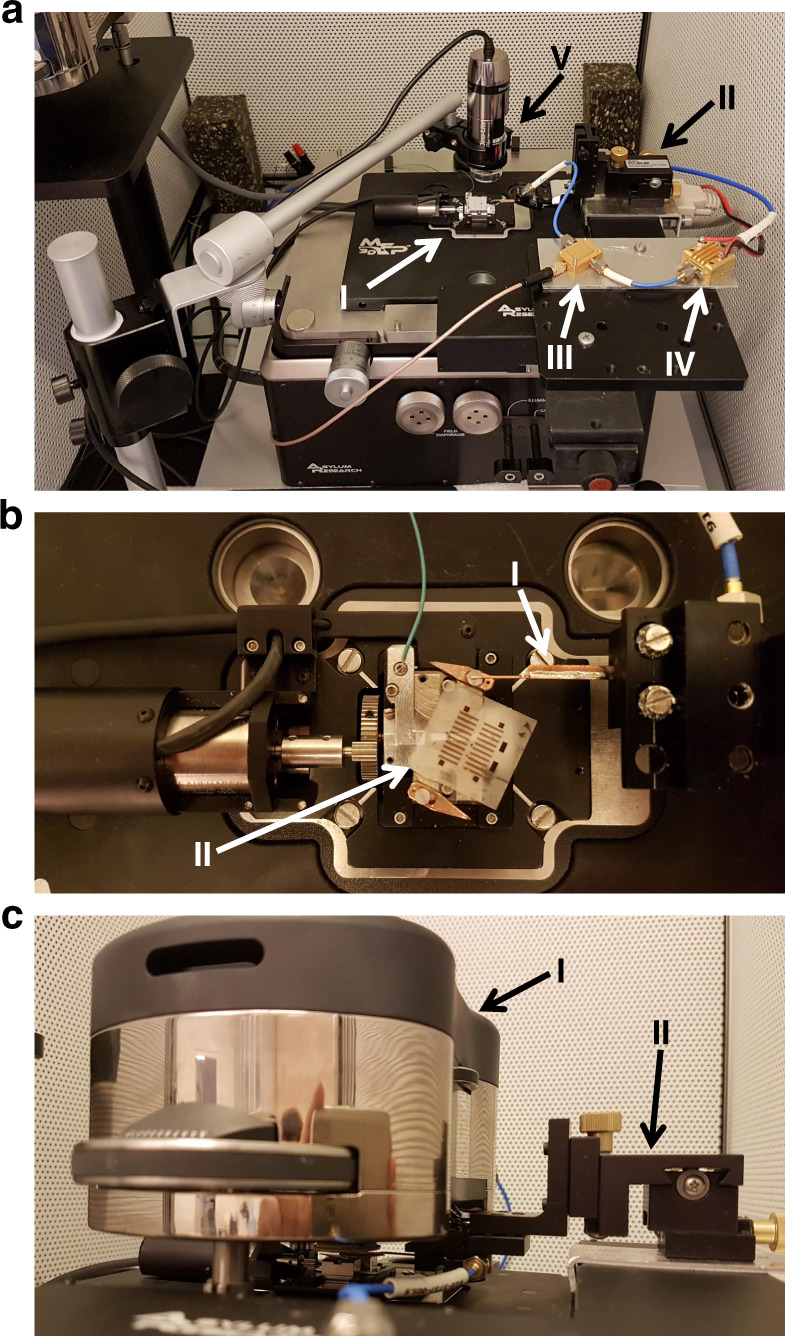
Developed MFM setup. **a** MFM setup consisting of I: an MFM stage with a version 2 variable field module (VFM2) and a microwave stage consisting of II: a micromanipulator with microwave probe; III: a bias-T; IV: an amplifier; and V: a microscope camera. **b** Top view of I: GSG microwave probe and II: mounted chip of devices on VFM2 with in-plane angle of *φ* = 24°; **c** side view of I: MFM head on top of the stage and II: micromanipulator while MFM scanning is in progress

Figure 3(a) shows our MFM system, an MFP-3D-SA^41^ from Asylum Research (an Oxford Instruments company), to which we added a microwave probe station^46,47^ to give electrical and microwave access to the devices under MFM scanning. Owing to space limitations, especially between the MFM head and the sample surface, it was geometrically difficult to provide electrical access to the device and a stable connection for transferring the microwave signals generated by the devices to be measured by the spectrum analyzer.

We first designed and built a holder for the XYZ micromanipulator and mounted it on the L-shaped slider of the MFM stage. Then, a GR-style nonmagnetic microwave probe from GGB was connected to the micromanipulator. Figure 3(b) shows a customized version of this probe with an extended co-axial line to reach the waveguides of the device we want to measure and simultaneously scan. In designing this probe, we tried to achieve the minimum thickness and height so that the probe, as well as its connection port to the RF cable, would fit in the limited space under the head of the MFM system without making contact and/or interrupting the scanning process. Outside of this limited region, we connected a transmission line for DC and microwave measurements.

Since we want to perform MFM and our samples are dependent on the applied magnetic field, we added a version 2 variable field module (VFM2)^48^ to the stage of the system (Fig. 3(b)). VFM2 uses permanent magnets to apply in-plane magnetic fields to our devices. The sample is then mounted on top of this module between the poles to achieve a uniform in-plane field. Moreover, we used a microscope to precisely make contact between the microwave probe and the sample. In addition, for the MFM process, we fabricated a customized high-resolution MFM probe using the process described in ref. ^42^.

**Fig. 4:**
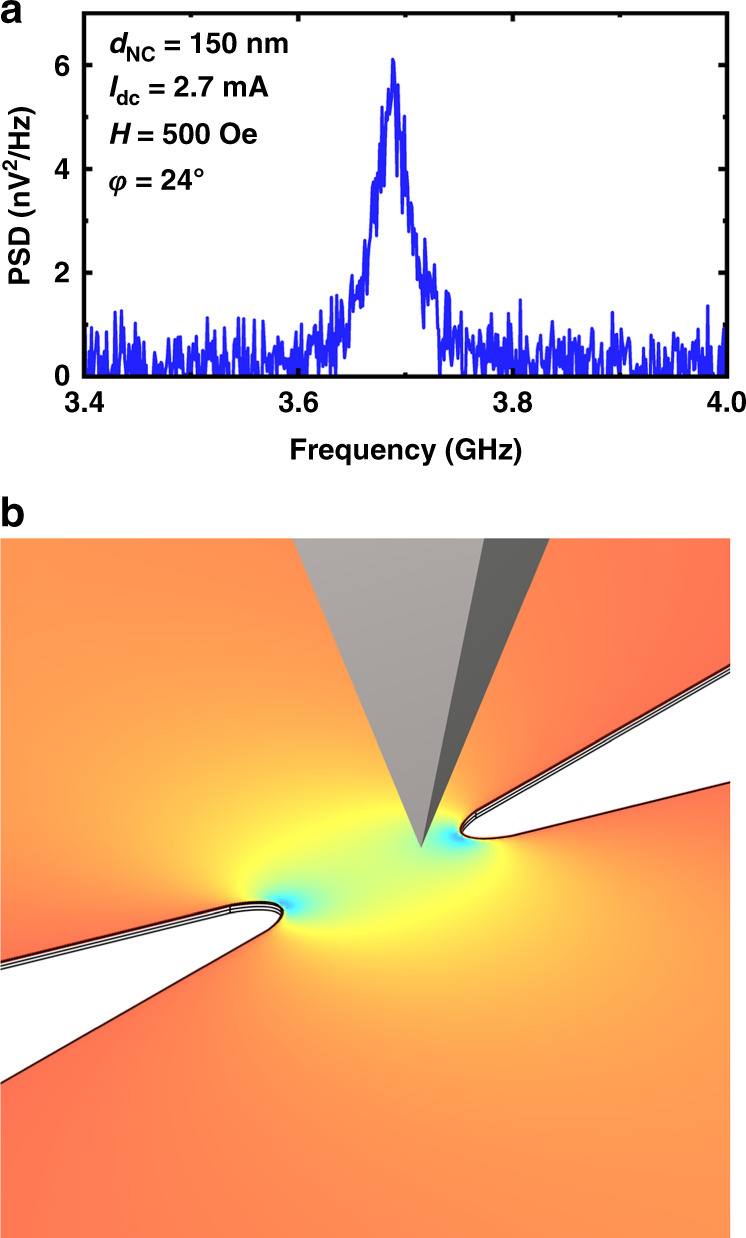
Output spectrum of operational SHNO and 3D simulation of MFM process. **a** Output spectrum of a nanoconstriction-based SHNO with a constriction width of *d*_NC_ = 150 nm and *I*_dc_ = 2.7 mA under an external field of *H* = 500 Oe with an in-plane angle of *φ* = 24°. **b** 3D plot of a simulated nanoconstriction-based SHNO with *d*_NC_ = 300 nm showing the *x*-component of *H*_Oe_ (with the same range as that shown in Fig. 2(b)) at the top of the Pt layer for *I*_dc_ = 6 mA, where a tetrahedral-shaped Co magnetic force microscopy tip with 10 nm spatial MFM resolution scans the operational device

Figure 3(b) shows a chip of 20 nanoconstriction-based SHNOs (with longer waveguides, as described in the previous section), which was mounted on top of VFM2 to apply an external in-plane magnetic field. For a consistent measurement, the device to be measured should be located exactly in the center between the poles of the magnets. At this position,we have a uniform field with the magnitude we set in the software. Furthermore, to apply the field at a certain angle, it is possible to rotate the chip and then adjust the angle, making contact with the microwave probe with the help of the microscope camera. We probed the device using the microwave probe, and then the MFM head was mounted (Fig. 3(c)). After calibrating and positioning the high-resolution MFM probe, we began the microwave measurements and then MFM scans on SHNOs with constriction widths ranging from 80 to 300 nm. To excite the magnetization of the mounted SHNO device, we applied an external in-plane magnetic field and allowed DC current to flow into the devices. Figure 4(a) displays the output power spectral density (PSD) of a nanoconstriction-based SHNO with a constriction width of *d*_NC_ = 150 nm and *I*_dc_ = 2.7 mA at an external field of H = 500 Oe with an in-plane angle of *φ* = 24° under current sufficient to induce auto-oscillations.

### Magnetic force microscopy of Spin Hall nano-oscillator

**Fig. 5:**
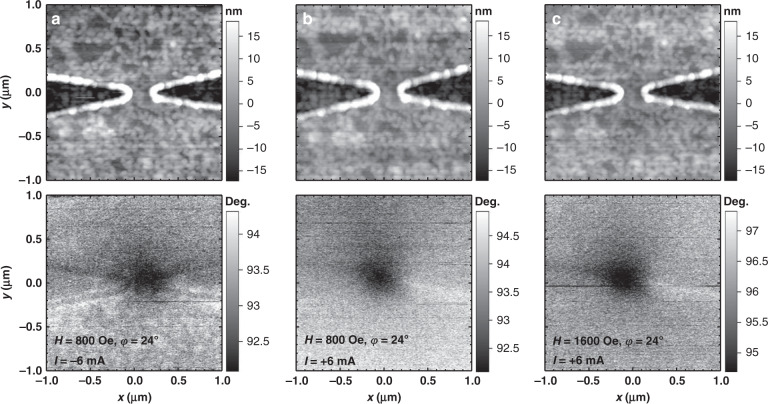
AFM and MFM results. AFM of a nanoconstriction-based SHNO with a constriction width of *d*_NC_ = 300 nm and the corresponding MFM below at applied fields and input currents of **a** *H* = 800 Oe and *I*_dc_ = −6 mA, **b** *H* = 800 Oe and *I*_dc_ = +6 mA, and **c** *H* = 1600 Oe and *I*_dc_ = +6 mA. The in-plane angle of the external field for all results is *φ* = 24°

Figure 4(b) shows a 3D plot of a simulated nanoconstriction-based SHNO with *d*_NC_ = 300 nm; the colors depict the strength of the *x*-component of *H*_Oe_ at the top of the Pt layer for *I*_dc_ = 6 mA, where a Co spike on a pyramidal magnetic force microscopy tip with 10 nm spatial MFM resolution scanned the operational nano-oscillator. Accordingly, we experimentally performed MFM on nanoconstriction-based SHNOs. Figure 5 shows the outputs of atomic force microscopy (AFM) and the corresponding phase shift data for the same area from the MFM lift pass below. The device is a nanoconstriction-based SHNO with a constriction width of *d*_NC_ = 300 nm under in-plane applied fields and input currents of (a) *H* = 800 Oe and *I*_dc_ = −6 mA, (b) *H* = 800 Oe and *I*_dc_ = +6 mA, and (c) *H* = 1600 Oe and *I*_dc_ = +6 mA. Additionally, the in-plane angle of the external field for all results is *φ* = 24°.

We consider the dark part of the MFM outputs (Fig. 5) to be the result of high current density, which in agreement with the simulation (Fig. 2(b)) induces an Oe field that can be recognized using the MFM technique. The devices do not show this feature in the absence of a magnetic field. We do not consider it to be magnetization oscillations because the output frequency of this type of device is in the range of 3–9 GHz, while the tapping frequency of this MFM scanner is on the order of kHz. This large difference makes it impossible to detect individual oscillations using this system. However, we do expect a system with a faster tapping mode to be able to scan the magnetization oscillations of magnetic nanodevices on the basis of the method we developed.

We presented a new method for probing the spatial profile of an operational magnetic nanodevice using magnetic force microscopy (MFM). We extended the MFM system by adding a microwave probe station to provide simultaneous electrical and microwave contact with our fabricated devices during the MFM process. Furthermore, because of the limited space under the MFM head, special devices with longer waveguides were designed and fabricated to ensure stable contact between the device and the microwave probe. Using this extended MFM system, we imaged operational nanoconstriction-based nano-oscillators.

The experimental results show that this method is indeed useful for extracting the spatial profile of an operational magnetic nanodevice. Therefore, developing the method as well as studying new devices are potential opportunities. In a future studies, a quantitative magnetic force microscope (e.g., the lab-built qMFM used in the study of ref. ^49^) can be developed by adding a similar electrical microwave probe station capable of scanning the operational SHNO. This would facilitate the comparison of results and the extraction of more data from our magnetic imaging. The results from the system that we used are not quantitative, so we are not able to compare different MFM scans.

## Materials and methods

### Sample fabrication

To fabricate our samples, 5 nm thick permalloy (Ni_80_Fe_20_; Py) and then 6 nm thick Pt were magnetron sputtered in a system with a base pressure lower than 3 × 10^−8^ Torr at room temperature onto an 18 × 18 mm piece of sapphire C-plane substrate and covered with 5 nm SiO_2_ in situ to prevent the oxidation of the permalloy layer. The layers were then patterned into 4 × 12 µm rectangles with two 50 nm tip radius indentations forming nanoconstrictions with nominal widths of *d*_NC_ = 80–300 nm by e-beam lithography and subsequent argon ion milling using a negative e-beam resist as the etching mask. Ultrasmall constriction widths were obtained by taking advantage of ion beam milling at an angle of 45° with respect to the film normal and the associated lateral erosion of the etching mask. Then, a long coplanar waveguide that provided electrical contact was connected to the nanoconstriction by optical lithography followed by a lift-off process of 980 nm of copper and 20 nm of gold.

### Electrical microwave probe station

To obtain electrical and microwave access to the nanodevice and then perform measurements, we used a custom-built probe station, where we mounted the sample with a fixed in-plane angle on top of a variable field module v.2 (VFM2)^48^. VFM2 uses permanent magnets (rather than electromagnetic coils) to avoid heating during ramping of the field and the associated drift and can apply in-plane magnetic fields of more than ±0.8 Tesla (8000 G) while offering a ∼1 G field resolution.

Moreover, a microscope was needed to make precise contact between the microwave probe and the sample. Therefore, for the vision part of the sample holder, we employed a Dino-Lite AD7013MZT digital hand-held microscope camera with a 5 megapixel sensor and an adjustable polarizer with up to x240 magnification on a fine-focus adjustment stand.

A direct electric current was applied to the devices through a high-frequency bias-T, and the resulting rf oscillations were amplified by a low-noise amplifier and recorded with a high-frequency spectrum analyzer (SA) using a low-resolution bandwidth of 1 MHz. The auto-oscillations were detected electrically in the tens of devices measured, and the results were reproducible from device to device.

### Simulation of the current density and its induced magnetic field

To investigate how the current redistributes in a nanoconstriction-based SHNO device and to calculate the induced Oersted field, calculations of the current flow in each layer of the SHNO were carried out using COMSOL Multiphysics^®^ (www.COMSOL.com) simulation software with a detailed three-dimensional finite-element model of the SHNO. We first built the structure of the device and then specified the materials and conditions of each domain. By defining the input and output of the current flow in the device structure, we then simulated and calculated the desired parameters.

### Magnetic force microscopy

We employed a magnetic force microscope, an MFP-3D-SA^41^ from Asylum Research (an Oxford Instruments company), and added a customized microwave probe station to this system. For MFM scanning, we used a standard cantilever for MFM measurements. Therefore, we had an MFM coating on the Olympus OMCL-AC240TS cantilever. In addition, to obtain a high-resolution MFM process, we fabricated a customized MFM probe using the process described in ref. ^42^ with a length of 1 µm, a diameter of 150 nm, and a cone-shaped head.
